# Anæsthetics in the Austrian Army

**Published:** 1856-03

**Authors:** 


*Anæsthetics in the Austrian Army.*—A circular has just been issued, ordering that in future the army medical officers shall always employ, for the purpose of inducing anæsthesia a mixture consisting of one part chloroform and nine parts ether, this being the proportion long employed by Dr. Weiger, a Vienna dentist.—*Med. Times and Gaz.*, Nov. 17, 1855.

